# The combined assessment of p16^INK4a^ and Mib/Ki-67 in oral squamous cell carcinoma

**DOI:** 10.3389/fonc.2024.1493281

**Published:** 2024-11-27

**Authors:** Maximilian Richter, Christian Doll, Friedrich Mrosk, Elena Hofmann, Steffen Koerdt, Max Heiland, Konrad Neumann, Marcus Beck, Steffen Dommerich, Korinna Jöhrens, Jan-Dirk Raguse

**Affiliations:** ^1^ Charité - Universitätsmedizin Berlin, corporate member of Freie Universität Berlin and Humboldt-Universität zu Berlin, Department of Oral and Maxillofacial Surgery, Berlin, Germany; ^2^ Berlin Institute of Health at Charité – Universitätsmedizin Berlin, BIH Biomedical Innovation Academy, BIH Charité Junior Clinician Scientist Program, Berlin, Germany; ^3^ Charité - Universitätsmedizin Berlin, Corporate Member of Freie Universität Berlin and Humboldt-Universität zu Berlin, Institute of Biometry and Clinical Epidemiology, Berlin, Germany; ^4^ Charité - Universitätsmedizin Berlin, Corporate Member of Freie Universität Berlin and Humboldt-Universität zu Berlin, Department of Radiooncology and Radiotherapy, Berlin, Germany; ^5^ Charité - Universitätsmedizin Berlin, Corporate Member of Freie Universität Berlin and Humboldt-Universität zu Berlin, Department of Otorhinolaryngology, Berlin, Germany; ^6^ Institute of Pathology, Klinikum Chemnitz, Chemnitz, Germany; ^7^ Department of Oral and Maxillofacial Surgery, Fachklinik Hornheide, Münster, Germany

**Keywords:** oral squamous cell carcinoma, OSCC, prognostic marker, p16, Ki-67, MIB

## Abstract

**Objective:**

Despite numerous studies addressing the impact of p16^INK4a^ in oral squamous cell carcinoma (OSCC), consistent data regarding survival and tumor proliferation behavior are lacking. Although some authors investigate both p16^INK4a^ and Mib/Ki-67 in their cohorts, direct correlations are consistently missing. The aim of this study was to investigate the combined influence of p16^INK4a^ and Mib/Ki-67 status on prognosis in OSCC.

**Materials and methods:**

Clinical data of all patients diagnosed with OSCC and treated curatively between 2005 and 2011 were collected retrospectively. Tissue microarrays of formalin-fixed paraffin-embedded specimens were stained for p16^INK4a^ and Mib/Ki-67 using the CINtec Histology V-Kit or MIB-1 antibody and correlated with the clinical outcome.

**Results:**

A total of 316 patients, with a mean age of 61.7 years were included. Tumor tissues that were tested p16^INK4a^ positive with low Mib/Ki-67 expression demonstrated a remarkable 5-year survival rate of 83% with an improved RFS compared to all other subgroups (p=0.034; p=0.017; p=0.026) and an improved OS compared to those with high Mib/Ki-67 expression (p=0.026; p=0.020). Cox regression identified the combined p16^INK4a^ and Mib/Ki-67 status as a risk factor on OS (HR 6.25; CI1.26-31.0; p=0.025) and RFS (HR 5.88; CI1.19-29.20; p=0.030).

**Conclusion:**

These results underscore the importance of a combined assessment of p16^INK4a^ and Mib/Ki-67 in evaluating the prognosis of OSCC, leading to the identification of distinct subgroups that may serve as risk factors for treatment stratification.

## Introduction

Oral squamous cell carcinoma (OSCC) is a form of epithelial cancer that originates from the mucosal surface of the mouth and represents the most prevalent type of oral cancer (1). Globally, 389,846 new cases of oral and lip carcinomas were diagnosed in 2022, accounting for approximately 2% of all malignant tumors (2). While developing countries such as India and Sri Lanka exhibit decreasing incidence rates, nations with historically low disease rates have observed an increase in incidence, placing significant strain on their healthcare systems (3).

In addition to well-established risk factors like tobacco use and alcohol consumption (4), the role of human papillomavirus (HPV) and/or p16^INK4a^ status remains poorly understood and requires further investigation. A deeper understanding of these risk factors could potentially lead to optimized risk-adjusted treatments and improved outcomes.

Given the context of low survival rates, factors such as tumor size and depth of invasion are long known prognostic indicators (5, 6). Furthermore, the impact of cell proliferation has been studied extensively by various authors. Expression of Ki-67, a widely accepted and practical tool for assessing the proportion of proliferating cells, was initially described in 1983 (7). Located on Chromosome 10, Ki-67 plays a major role in mitosis and is present in all proliferating cells, while being absent in resting cells (8). A meta-analysis published in 2016 indicated an adverse impact on overall survival (OS) and disease-free survival in OSCC with high Ki-67 positivity (9). Given the limited sample sizes and heterogeneity of the included studies, particularly regarding the use of different antibodies/methods and encompassing diverse ethnicities, further investigations are needed (9).

In contrast, there is a discrepancy in data regarding the influence of p16^INK4a^ positivity in OSCC. Acting as a cell cycle protein, increased expression of p16^INK4a^ naturally inhibits cyclin-dependent kinases, thereby inducing cellular senescence (10). While a beneficial prognostic effect of increased p16^INK4a^ has been demonstrated in oropharyngeal squamous cell carcinoma (OPSCC) (11), alongside an association with HPV status (12), this correlation does not seem to apply to OSCC, with most authors reported no influence of p16^INK4a^ status on survival (13, 14).

The integration of a combined assessment of p16^INK4a^ and Mib/Ki-67 status, known as dual-staining, has been implemented in the early detection of pathologies in cytological sections of the cervix uteri (15). The distinctive decoupling, which demonstrates a high proliferation rate despite increased p16^INK4a^ expression, has led to an improved identification of cervical intraepithelial neoplasia (15). Similar findings have been observed in tumors of the head and neck region, suggesting an enhanced detection of the malignant transformation of oral submucous fibrosis (16). Nevertheless, a significant impact on survival has not been confirmed for OSCC (17).

The aim of this study was to evaluate the prognostic role of combined p16^INK4a^ and Mib/Ki-67 assessment in a substantial cohort of patients with OSCC.

## Patients and methods

### Ethics statement

The Ethics Committee of the Faculty of Medicine Charité - Universitätsmedizin Berlin approved this study (EA2/028/15).

### Data collection

Clinical data of all patients diagnosed with curatively treated OSCC between 2005 and 2011 were collected retrospectively including TNM stage, age, gender, survival time and treatment regime. In case of a surgical treatment further information especially the pathological TNM was recorded.

OS was defined as the time span between the date of initial diagnosis (or, if not available, the date of therapy initiation) and the date of death or was censored at the date of the last contact.

Recurrence-free survival (RFS) was described as the time span from the end of therapy until the occurrence of a recurrence or until the time of death. In contrast, tumors that recurred within the first six months after surgery with R1/Rx status or primary radio(chemo)therapy were classified as residual tumors and were excluded from RFS analysis.

Patients receiving palliative care, those who underwent only a single induction therapy, those who received no therapy, or those who discontinued radiochemotherapy were not considered.

### Immunohistochemical staining

Immunohistochemical analyses were performed using tissue microarrays (TMAs). Initially, archived formalin-fixed paraffin-embedded specimens were retrieved and marked based on the hematoxylin-eosin-stained sections of the tumor. Representative sections were transferred using 1 or 2 punches (1.2 mm in diameter), depending on tumor size. In total, 15 TMAs were produced, each containing 66 punches. The subsequent evaluation of the immunohistochemical stainings, as described below, was carried out by two independent examiners.

### p16^INK4a^ analysis

The CINtec Histology V-Kit (Roche, Switzerland), an antibody against the E6H4 epitope, was used for immunohistochemical staining. The staining process was fully automated using the Ultraview DAB procedure of the VENTANA BenchMark ULTRA staining system (Roche, Switzerland). In accordance with Prigge et al., tumors were considered p16^INK4a^ positive if at least 70% of tumor cells exhibit cytoplasmic and/or nuclear expression and demonstrate at least moderate staining intensity (18).

### Mib/Ki-67 analysis

Following cell conditioning with ULTRA Cell Conditioning Solution (Ventana, USA; Roche, Switzerland), immunohistochemical staining was performed using the MIB-1 antibody (Agilent, USA), a monoclonal antibody for a 1002 bp fragment of the Ki-67 cDNA, at a ratio of 1:50. The staining process was fully automated using the Ultraview DAB procedure of the VENTANA BenchMark ULTRA staining system (Roche, Switzerland). Referring to the publication by Lange et al., a threshold value of ≥37% was defined as Mib/Ki-67 positive (19).

### HPV DNA analysis

Samples defined p16^INK4a^ positive were examined for the presence of HPV DNA. DNA extraction was performed on formalin-fixed samples using the Maxwell RSC DNA FFPE Kit (Promega, Germany). For DNA amplification, a polymerase chain reaction (PCR) was conducted (denaturation at 95°C for 5 minutes, amplification with a total of 45 cycles, and elongation at 72°C for 7 minutes). HPV typing was then carried out using the HPV 3.5 LCD-Array Kit (Chipron, Germany; TIB Molbiol, Germany) to detect specific regions of the L1 gene. This method allows for the identification of HPV types 6, 11, 16, 18, 31, 33, 35, 39, 42, 44, 45, 51, 52, 53, 54, 56, 58, 59, 61, 62, 66, 67, 68, 70, 72, 73, 81, 82, 83, 84, 90, and 91.

### Statistical analysis

Data were collected using Microsoft Excel version 15 (Microsoft Corporation, Redmond, WA, USA) and analyzed with IBM SPSS Statistics version 27 and R version 4.0.3 (20). Categorical variables were presented as frequency and percentage, while continuous variables were expressed as mean values. Frequencies of categorical or ordinal variables were compared using the chi-square test, or Fisher’s exact test when expected frequencies were less than 5.

Overall survival (OS) and relapse-free survival (RFS) were analyzed using the Kaplan-Meier method and compared with the log-rank test. Multivariate Cox regression analysis was conducted to evaluate combined p16^INK4a^ and Mib status as risk factors. All p-values are exploratory and have not been adjusted for multiple comparisons. Statistical significance was determined at a threshold of α=0.05, with p-values below this threshold considered significant.

## Results

A total of 316 patients (female: 110 (34.8%), male: 206 (65.2%)) met the inclusion criteria with an average age of 61.7 years (SD ±12.0 years; range 27-96 years) at initial diagnosis. The therapies included surgery without an adjuvant therapy (n=201, 63.6%), followed by surgery with adjuvant therapy (n=75; 23.7%) and primary radio(chemo)therapy (n=40; 12.7%).

The mean OS in this cohort was 79.9 months (CI: 73.9-85.9 months), with a 5-year survival rate of 59%. RFS (as defined above) was 66.3 months (CI: 60.2-72.4 months).

### p16^INK4a^ analysis

Only 21 out of 316 tumors (6.6%) tested positive for p16^INK4a^. Patients with p16^INK4a^ positive and p16^INK4a^ negative tumors exhibited no differences in the clinical parameters examined (**Table 1**). Survival analysis revealed better OS for patients with p16^INK4a^ positive tumors, with a 10-year survival rate of 60% (OS: 87.6 months; CI: 63.7-111.4), compared to patients with p16^INK4a^ negative tumors, who demonstrated a 10-year survival rate of 39% (OS: 79.2 months; CI: 73.1-85.4). However, this difference was not statistically significant (p=0.359). Similarly, in terms of RFS, we observed comparable trends, with a better survival of 80.6 months in p16^INK4a^ positive tumors (compared to 65.2 months in p16^INK4a^ negative tumors, p=0.194).

**Table 1:** Clinical characteristics in patients with p16^INK4a^ positive and p16^INK4a^ negative tumors.

	p16+	p16-	p-value
total	21 (6.6%)	295 (93.4%)	
Mean age at initial diagnosis	59.4 years	61.9 years	0.364
Mean age at surgery	59.9 years	61.7 years	0.539
Therapy (n=316)			
Surgery without adjuvant therapy	13 (4.1%)	188 (59.5%)	0.624
Surgery with adjuvant therapy	4 (1.3%)	71 (22.5%)	
Primary radio (chemo)therapy	4 (1.3%)	36 (11.4%)	
Sex (n=316)			
male	16 (5.1%)	190 (60.1%)	0.197
female	5 (1.6%)	105 (33.2%)	
History of alcohol (n= 280)			
Yes	17 (6.1%)	195 (69.6%)	0.116
No	2 (0.7%)	66 (23.6%)	
History of smoking (n=282)			
Yes	17 (6.0%)	194 (68.8%)	0.21
No	3 (1.1%)	68 (24.1%)	
Pathological UICC (n=264)			
I	5 (1.9%)	80 (30.3%)	0.815
II	6 (2.3%)	66 (25.0%)	
III	2 (0.8%)	47 (17.8%)	
IV	4 (1.5%)	54 (20.5%)	
Grading (n=287)			
1	0	21 (7.3%)	0.051
2	12 (4.2%)	219 (76.3%)	
3	5 (1.7%)	30 (10.5%)	
Bone infiltration (n=276)			
Yes	6 (2.2%)	53 (19.2%)	0.129
No	11 (4.0%)	206 (74.6%)	
Extracapsular spread (n=254)			
Yes	2 (0.8%)	33 (13.0%)	0.803
No	15 (5.9%)	204 (80.3%)	

For the analysis of HPV DNA, 18 of 21 p16^INK4a^ positive samples were available. Amplification could not be detected in 4 cases. In 5 of the remaining 14 p16^INK4a^ positive samples (35.7%), HPV was successfully detected (in all cases HPV 16 DNA).

When analyzing OS/RFS regarding type of therapy, we found no statistically significant differences between p16^INK4a^ positive and p16^INK4a^ negative tumors in patients who received surgery without adjuvant therapy (p=0.532/p=0.430), surgery with adjuvant therapy (p=0.198/0.152) or primary radio(chemo)therapy (p=0.544/p=0.433).

### Mib/Ki-67 analysis

The mean Mib/Ki-67 expression was 28.3% (SD ±17.3%) tested in 316 tumors.

When analyzing the mean Mib/Ki-67 positivity depending on the grading we found significant differences between G1 (Mib/Ki-67 expression: 11.8%) and G2 tumors (Mib/Ki-67 expression: 29.0%, p<0.001) and between G1 and G3 tumors (Mib/Ki-67 expression: 33.0%, p<0.001).

However, in the entire cohort, no cut-off value could be identified that showed a significant influence on the OS or RFS. When performing survival analysis for respective therapy regiments with a cut-off value of 37%, we found no significant differences in OS or RFS between Mib/Ki-67 positive and negative tumors in patients who underwent surgery without adjuvant therapy (p=0.061/p=0.103), surgery with adjuvant therapy (p=0.857/p=0.944) or primary radio(chemo)therapy (p=0.471/p=0.311).

### Combined assessment of p16^INK4a^ and Mib/Ki-67

Next, subanalyses were conducted for the combined assessment of p16^INK4a^ and Mib/Ki-67.

The mean Mib/Ki-67 positivity in the 21 patients that tested positive for p16^INK4a^ was 32.5% (SD ±17.4%) compared to 28.0% (SD ±17.2%) in the 295 patients with p16^INK4a^ negative tumors (p=0.240). No differences in proliferation rates were found between tumors with predominantly nuclear p16^INK4a^ expression (n=11) and those with primarily cytoplasmic or balanced expression (n=10; p=0.194).

A cut-off value in the Mib/Ki-67 immunohistochemistry of 37% based on Lange et al. (19) was selected to divide the cohort into four subgroups: p16^INK4a^ positive/Mib positive (p16+/Mib+), p16^INK4a^ positive/Mib negative (p16+/Mib-), p16^INK4a^ negative/Mib negative (p16-/Mib-) and p16^INK4a^ negative/Mib positive (p16-/Mib+) tumors.

Regarding clinical characteristics or HPV DNA detection we found no significant differences between p16+/Mib+ and p16+/Mib- tumors (**Table 2**).

**Table 2:** Clinical characteristics in patients with p16+/Mib+ and p16+/Mib- tumors.

	p16+/Mib+	p16+/Mib-	p-value
total	8 (38.1%)	13 (61.9%)	
Mean age at initial diagnosis	56.9 years	63.6 years	0.364
Sex (n=21)			
male	7 (33.3%)	9 (42.9%)	0.606
female	1 (4.8%)	4 (19.0%)	
History of alcohol (n= 19)			
Yes	7 (36.8%)	10 (52.6%)	0.678
No	1 (5.3%)	1 (5.3%)	
History of smoking (n=20)			
Yes	7 (35.0%)	10 (50.0%)	1.0
No	1 (5.0%)	2 (10.0%)	
UICC (n=20)			
I	1 (5.0%)	4 (20.0%)	0.062
II	0	6 (30.0%)	
III	1 (5.0%)	0	
IVa	4 (20.0%)	3 (15.0%)	
IVb	1 (5.0%)	0	
Grading (n=17)			
1	0	0	0.101
2	3 (17.6%)	9 (52.9%)	
3	4 (23.5%)	1 (5.9%)	
Bone infiltration (n=17)			
Yes	2 (11.8%)	4 (23.5%)	0.584
No	2 (11.8%)	9 (52.9%)	
Extracapsular spread (n=17)			
Yes	1 (5.9%)	1 (5.9%)	0.426
No	3 (17.6%)	12 (70.6%)	
HPV DNA (n=14)			
Positive	2 (14.3%)	3 (21.4%)	1.0
Negative	4 (28.6%)	5 (35.7%)	

**Figure 1:**
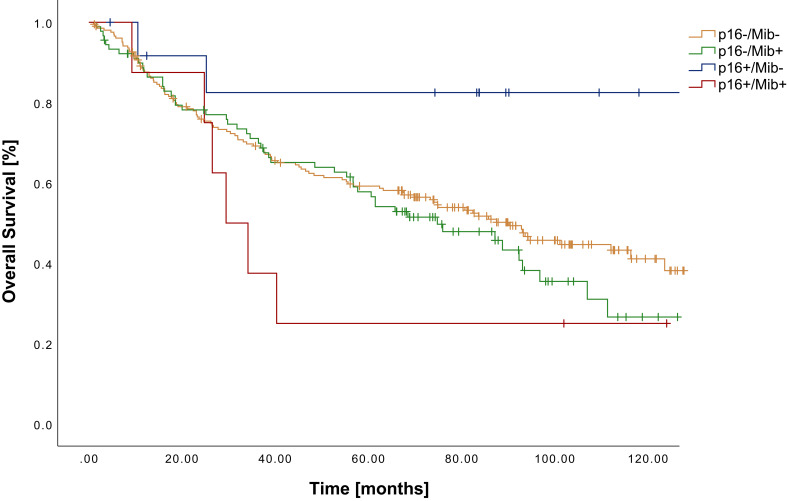
Kaplan-Meier curves showing OS of patients with OSCC in relation to the combined p16^INK4a^ (cut-off ≥70%) and Mib/Ki-67 status (cut-off ≥37%).

We observed a significant superiority of p16+/Mib- tumors compared to p16-/Mib+ (p=0.026) and p16+/Mib+ (p=0.020) leading to a 5-year survival rate of 83%. In contrast, p16+/Mib+ showed a 5-year survival rate of 25%, which was even lower than that of p16-/Mib- (59%) or p16-/Mib+ tumors (58%) (**Figure 1**; **Table 3**). The combined p16/Mib status emerged as a risk factor for OS in the Cox regression, with a hazard ratio (HR) of 6.25 (CI: 1.26-31.0) in the group of p16+/Mib+ tumors compared to p16+/Mib- tumors (p=0.025) (**Table 4**).

**Table 3:** OS in relation to the subgrouping of patients according to the combined assessment of p16^INK4a^ and Mib/Ki-67.

	n	OS [months]	5 YSR	p16-/Mib-	p16-/Mib+	p16+/Mib-	p16+/Mib+
p16-/Mib-	205	81.0	59%		*p=0.302*	*p=0.058*	*p=0.194*
p16-/Mib+	90	73.3	58%	p=0.302		***p=0.026***	*p=0.311*
p16+/Mib-	13	112.3	83%	p=0.058	**p=0.026**		***p=0.020***
p16+/Mib+	8	51.5	25%	p=0.194	p=0.311	**p=0.020**	

Bold values indicate statistically significant results (p<0.05).The table design results in repeated p-values, which are highlighted in gray.

**Table 4:** Cox regression for OS according to the combined assessment of p16^INK4a^ and Mib/Ki-67.

	No. of events/No. of patients (event rate)	Hazard Ratio	95%-Confidence interval	p-value
p16+/Mib-	2/13 (15.4%)	1.0		
p16+/Mib+	6/8 (75.0%)	6.25	1.26 - 31.01	**0.025**
p16-/Mib-	103/205 (50.2%)	3.57	0.88 – 14.47	0.075
p16-/Mib+	50/90 (55.6%)	4.24	1.03 – 17.45	**0.045**

Bold values indicate statistically significant results (p<0.05).

**Figure 2:**
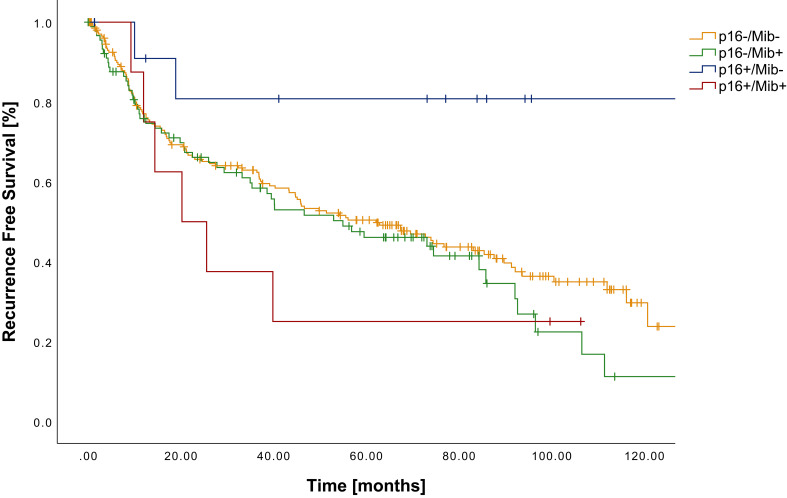
Kaplan-Meier curves showing RFS of patients with OSCC in relation to the combined p16^INK4a^ (cut-off ≥70%) and Mib/Ki-67 status (cut-off ≥37%).

Similar findings were evident in RFS with a statistically significant better outcome in patients with p16+/Mib- tumors compared to p16+/Mib+, p16-/Mib+ and p16-/Mib- tumors (p=0.026; p=0.017; p=0.034) (**Figure 2**). Mean RFS varied from 106.9 months (CI: 79.6-134.3 months) in p16+/Mib- to 41.7 months (CI: 15.2-68.3 months) in p16+/Mib+ tumors (**Table 5**). Cox regression showed a HR of 5.88 (CI: 1.19-29.20) in the group of p16+/Mib+ tumors compared to the p16+/Mib- tumors (p=0.030) (**Table 6**).

**Table 5:** RFS in relation to the subgrouping of patients according to the combined assessment of p16^INK4a^ and Mib/Ki-67.

	n	RFS [months]	p16-/Mib-	p16-/Mib+	p16+/Mib-	p16+/Mib+
p16-/Mib-	205	67.1		*p=0.314*	***p=0.034***	*p=0.368*
p16-/Mib+	90	58.8	p=0.314		***p=0.017***	*p=0.625*
p16+/Mib-	13	106.9	**p=0.034**	**p=0.017**		***p=0.026***
p16+/Mib+	8	41.7	p=0.368	p=0.625	**p=0.026**	

Bold values indicate statistically significant results (p<0.05).The table design results in repeated p-values, which are highlighted in gray.

**Table 6:** Cox regression for RFS according to the combined assessment of p16^INK4a^ and Mib/Ki-67.

	No. of events/No. of patients (event rate)	Hazard Ratio	95%-Confidence interval	p-value
p16+/Mib-	2/13 (15.4%)	1.0		
p16+/Mib+	6/8 (75.0%)	5.88	1.19 - 29.20	**0.030**
p16-/Mib-	114/205 (55.6%)	4.02	0.99 – 16.29	0.051
p16-/Mib+	53/90 (58.9%)	4.74	1.15 – 19.47	**0.031**

Bold values indicate statistically significant results (p<0.05).

## Discussion

Nowadays, the role of p16^INK4a^ as a predictor for prognosis in head and neck cancers, especially in OPSCC, is widely accepted (21, 22). However, in OSCC, its role remains unclear, leading to different approaches in implementing it into clinical routines.

Most studies report no significant influence of the p16^INK4a^ status on OS or RFS in OSCC (13, 23). However, there is a divergence of study results, with some authors suggesting a negative (24) or positive (25) prognostic impact. Many authors attribute these discrepancies to varied definitions of p16^INK4a^ positivity and small patient cohorts (26, 27). In our study, we applied a widely accepted definition in a large cohort of 316 patients with OSCC. Even within this sizable group, only 21 tumors (6.6%) tested positive for p16^INK4a^, demonstrating no significant influence on OS or RFS. In summary, our results concur with the reports that the singular p16^INK4a^ status has a minimal or likely absent influence in OSCC.

Numerous studies have investigated the impact of Mib/Ki-67 in OSCC. Comparable to our results, studies have shown a correlation between the proliferation rate and tumor grading, with higher rates of Mib/Ki-67 positive cells in poorly differentiated carcinomas (28). Similar associations are also found in premalignant stages (29). Furthermore, the heightened responsiveness of proliferating cells to radiation and chemotherapy has been consistently observed, correlating with increased tumor survival rates in cases with elevated Mib/Ki-67 levels (9). Nonetheless, our analysis revealed no influence of the Mib/Ki-67 status on OS or RFS (cut off 37%). The absence of a universally accepted cut-off value necessitates ongoing research efforts to harmonize the interpretation of Mib/Ki-67 immunohistochemical staining results (9). The validation of a consistent cut-off could enhance the reliability of Mib/Ki-67 as a prognostic marker, offering valuable insights into treatment response and aiding clinicians in refining therapeutic strategies for patients with OSCC.

The combined assessment of p16^INK4a^ and Mib/Ki-67, known as dual-staining, has been described already for pathologies of the cervix uteri. A large study by Schmidt et al. showed a significant superiority of the dual-staining compared to HPV testing alone in detection of cervical intraepithelial neoplasia (15). Furthermore, the authors delineated advantages in comparison to singular p16^INK4a^ staining, admitting benefits to the morphology independence of the dual-staining (15). Ziemke and Marquardt found a better specificity and positive predictive value of the dual-staining concerning the presence of intraepithelial neoplasia compared to cytology and HPV testing in low grade dysplasia of the cervix (30).

In neoplasm of the oral cavity there are only a few studies which examine dual-staining.

Bazarsad et al. highlighted the combined assessment of p16^INK4a^ and Mib/Ki-67 as a predictor for the malignant transformation of oral submucous fibrosis in a small collective of 36 patients (16). In context of OSCC, Reuschenbach et al. reported a slightly better survival in p16+/Mib- tumors; however, this finding did not reach statistical significance (17). As in our cohort, no association with HPV could be demonstrated (17). When comparing these results with our data, variations in the definitions of p16^INK4a^ or Mib/Ki-67 positivity must be acknowledged. However, there is a lack of other publications that include the combined assessment of p16^INK4a^ and Mib/Ki-67 regarding the prognosis of OSCC.

Our findings suggest that the role of p16^INK4a^ should be evaluated in the context of Mib/Ki-67 status, leading to entirely different subgroups of OSCC. We hypothesize that a combined assessment of p16^INK4a^ and Mib/Ki-67 could provide an explanation for the observed lack of influence when considering p16^INK4a^ status alone. Nevertheless, it is important to note the reduced statistical power of the analyses due to the small sample size of p16^INK4a^ positive tumors and the need for further investigations to understand the specific biological relationship between p16^INK4a^ and Mib/Ki-67 in OSCC.

In conclusion, the relation between p16^INK4a^ and Mib/Ki-67 must be reevaluated in other cohorts, particularly in those with a higher p16^INK4a^ prevalence, to potentially implement these findings into clinical practice.

## Data Availability

The original contributions presented in the study are included in the article/supplementary material. Further inquiries can be directed to the corresponding authors.
